# Limitations of MTT and MTS-Based Assays for Measurement of Antiproliferative Activity of Green Tea Polyphenols

**DOI:** 10.1371/journal.pone.0010202

**Published:** 2010-04-16

**Authors:** Piwen Wang, Susanne M. Henning, David Heber

**Affiliations:** Center for Human Nutrition, David Geffen School of Medicine, University of California Los Angeles, Los Angeles, California, United States of America; Virginia Commonwealth University, United States of America

## Abstract

**Background:**

The chemopreventive effect of green tea polyphenols, such as (-)-epigallocatechin-3-gallate (EGCG), has been well demonstrated in cell culture studies. However, a wide range of IC_50_ concentrations has been observed in published studies of the anti-proliferative activity of EGCG from different laboratories. Although the susceptibility to EGCG treatment is largely dependent on cancer cell type, the particular cell viability and proliferation assays utilized may significantly influence quantitative results reported in the literature.

**Methodology/Principal Findings:**

We compared five widely used methods to measure cell proliferation and viability after EGCG treatment using LNCaP prostate cancer cells and MCF-7 breast cancer cells. Both methods using dyes to quantify adenosine triphosphate (ATP) and deoxynucleic acid (DNA) showed accuracy in the measurement of viable cells when compared to trypan blue assay and results showed good linear correlation (r = 0.95). However, the use of MTT (3-(4,5-Dimethylthiazol-2-yl)-2,5-diphenyltetrazolium bromide) and MTS (3-(4,5-dimethylthiazol-2-yl)-5-(3-carboxymethoxyphenyl)-2-(4-sulfophenyl)-2H-tetrazolium) as indicators of metabolically active mitochondria overestimated the number of viable cells by comparison with the ATP, DNA, or trypan blue determinations. As a result, the observed IC_50_ concentration of EGCG was 2-fold higher using MTT and MTS compared to dyes quantifying ATP and DNA. In contrast, when cells were treated with apigenin MTT and MTS assays showed consistent results with ATP, DNA, or trypan blue assays.

**Conclusions/Significance:**

These results demonstrate that MTT and MTS -based assays will provide an underestimation of the anti-proliferative effect of EGCG, and suggest the importance of careful evaluation of the method for in vitro assessment of cell viability and proliferation depending on the chemical nature of botanical supplements.

## Introduction

Tea and tea polyphenols are promising chemopreventive agents against a variety of tumors, including lung, esophagus, stomach, liver, prostate, pancreas, colon, and breast [1]–[5]. The active constituents in green tea are believed to be polyphenols including (-)-epigallocatechin, (-)-epigallocatechin-3-gallate (EGCG), (-)-epicatechin, and (-)-epicatechin-3-gallate, with EGCG being the most abundant and possibly the most bioactive of the green tea polyphenols [6].

In vitro cell culture studies are valuable tools for screening of chemopreventive agents and provide preliminary data for in vivo studies. The anti-proliferative activity of EGCG has been demonstrated in various cancer cell lines, however, the half maximal inhibitory concentration (IC_50_) values from different studies vary greatly from tens to hundreds of µmol/L of EGCG [7]-[9]. The susceptibility to EGCG treatment is largely dependent on cell type. However the choice of method to evaluate viability and proliferation may also significantly influence the quantitative assessment of anti-proliferative activities of botanicals such as green tea.

Many methods have been developed to measure cell proliferation including those based on direct counting of viable cells, measurement of metabolic activity and cellular DNA content. The traditional cell counting methods such as trypan blue dye exclusion assay using a hemacytometer are simple and inexpensive but very time consuming and sometimes inaccurate [10]. Measurement of mitochondrial metabolic rate using MTT (3-(4,5-Dimethylthiazol-2-yl)-2,5-diphenyltetrazolium bromide) and MTS (3-(4,5-dimethylthiazol-2-yl)-5-(3-carboxymethoxyphenyl)-2-(4-sulfophenyl)-2H-tetrazolium) to indirectly reflect viable cell numbers has been widely applied [7]-[9], [11]. However, metabolic activity may be changed by different conditions or chemical treatments which can cause considerable variation in results reported from these assays [12], [13].

In the present study, we compared the results of the methods using trypan blue, MTT, MTS, as well as dyes quantifying ATP and DNA to determine the effect of EGCG and apigenin on cell viability and proliferation in LNCaP prostate cancer cells and MCF-7 breast cancer cells.

## Materials and Methods

### Chemicals and Reagents

EGCG (>95% purity), MTT (3-(4,5-Dimethylthiazol-2-yl)-2,5-diphenyltetrazolium bromide), and dimethyl sulfoxide (DMSO) were purchased from Sigma Chemicals (St Louis, MO). CellTiter 96® AQ_ueous_ One Solution Cell Proliferation Assay (MTS) kit and CellTiter-Glo® Luminescent Cell Viability Assay (ATP) kit were purchased from Promega Corporation (Madison, WI). CyQUANT® NF Cell Proliferation Assay (DNA) Kit was purchased from Invitrogen Corporation (Carlsbad, CA).

### Cell Line and Cell Culture

The human prostate adenocarcinoma LNCaP cells was obtained from ATCC (Chicago, IL), and cultured in RPMI 1640 medium with glutamine supplemented with 10% (v:v) of fetal bovine serum (FBS) (USA Scientific, Ocala, FL), 100 IU/ml of penicillin and 100 µg/ml of streptomycin (Invitrogen Inc, Carlsbad, CA). The MCF-7 cells were generously provided by the Chen Lab, City of Hope/Beckman Research Institute (Duarte, CA). The cells were transfected with aromatase gene [14]. The cells were cultured in MEM medium with glutamine containing 10% FBS, 1 mM sodium pyruvate, 10 IU/ml of penicillin and 10 µg/ml of streptomycin, and 100 µg/ml of G418. All the cells were cultured at 37 µC in a 5% CO_2_ incubator. At pH above 6.5 EGCG undergoes autoxidation and dimerization and forms hydrogen peroxide (H_2_O_2_) [15]. To minimize the H_2_O_2_ effect 50 units/ml of catalase was added to the medium prior to EGCG. In addition, another flavonoid apigenin was used as a control compound to validate the interference of EGCG with different assays. LNCaP cells (1×10^4^ per well on 96-well plate) were treated with either DMSO, 15 µmol/L or 30 µmol/L apigenin for 24 h, 48 h, or 72 h, and the viable cells were measured with the same assays as that for EGCG treated cells.

### Trypan Blue Exclusion Assay

LNCaP cells were seeded into 96-well plates at a density of 1×10^4^ per well (100 µl) and treated with the following: vehicle control (DMSO), EGCG at 40 and 80 µmol/L. MCF-7 cells were seeded into 96-well plates at a density of 0.8×10^4^ per well and treated with the following: vehicle control (DMSO), EGCG at 20 and 40 µmol/L. The cells were treated for 24 h, 48 h, or 72 h, and cell growth was photographed under microscope at each time point.

Cell viability was determined by trypan blue dye exclusion assay. Triplicate wells of viable cells for each concentration were counted on a hemacytometer after trypsinization. Each well had three repeats of counting. The experiment was repeated three times.

### MTT and MTS Assays

LNCaP cells were seeded into 96-well plates at a density of 1×10^4^ per well (100 µl) and treated with the following: vehicle control (DMSO), EGCG at 40 and 80 µmol/L. MCF-7 cells were seeded into 96-well plates at a density of 0.8×10^4^ per well and treated with the following: vehicle control (DMSO), EGCG at 20 and 40 µmol/L. Wells with serum free medium were used as negative control. The cells were treated for 24 h, 48 h, or 72 h.

For the MTT assay, at 3 h before each of the desired time points, 20 µl of MTT solution (5 mg/ml in PBS) was added into each well and cells were incubated at 37 µC for another 3 h. The medium was removed and 100 µl of DMSO was added into each well. The plate was gently rotated on an orbital shaker for 10 min to completely dissolve the precipitation. The absorbance was detected at 570 nm with a Microplate Reader (VersaMax, Molecular Devices, Sunnyvale, CA).

For the MTS assay, the CellTiter 96® AQ_ueous_ One Solution Cell Proliferation Assay kit was used following the manufacturer's instruction. Briefly, at 3 h before each of the desired time points, 10 µl of the MTS reagent was added into each well and cells were incubated at 37 µC for 3 h. The absorbance was detected at 490 nm with a Microplate Reader (VersaMAx, Molecular Devices). All the experiment was repeated three times.

### ATP and DNA Assays

LNCaP cells were seeded into 96-well opaque-walled plates at a density of 1×10^4^ per well (100 µl) and treated with the following: vehicle control (DMSO), EGCG at 10, 20, 40, 60, 80, 100, and 120 µmol/L; MCF-7 cells were seeded into 96-well plates at a density of 0.8×10^4^ per well and treated with the following: vehicle control (DMSO), EGCG at 10, 20, 30, 40, 60, and 80 µmol/L for 24 h, 48 h, or 72 h. Wells with serum free medium were used as negative control.

The CellTiter-Glo® Luminescent Cell Viability Assay kit was used for ATP assay following the manufacturer's instruction. Briefly, the assay buffer and substrate were equilibrated to room temperature, and the buffer was transferred to and gently mixed with the substrate to obtain a homogeneous solution. After a 30 min equilibration of the cell plate to room temperature, 100 µl of the assay reagent was added into each well and the content was mixed for 2 min on an orbital shaker to induce cell lysis. After 10 min incubation in room temperature, the luminescence was read on a Microplate Reader (SpectraMax GeminiEM, Molecular Devices, Sunnyvale, CA).

The CyQUANT® NF Cell Proliferation Assay kit was used for DNA assay following the manufacturer's instruction. Briefly, the cell growth medium was removed and 100 µl of 1x lysis/dye binding solution was added into each well. The plate was incubated at 37 µC for 50 min, and the fluorescence intensity was measured on a Microplate Reader (SpectraMax GEminiEM, Molecular Devices) with a wavelength of 485 nm for excitation and 530 nm for emission. All the experiments were repeated three times.

### Statistical Analysis

SPSS (Version 18.0, Chicago, IL) was used for all statistical analyses. Mean value and standard deviation were calculated using descriptive statistics. IC_50_ values were calculated by Probit regression. Comparison of means was performed by one-way analysis of variance with Tukey's posttest. Comparison of assays was made by correlation and linear regression analysis. Differences were considered significant if P<0.05.

## Results

This study has demonstrated a twofold underestimation of the anti-proliferative effect of EGCG when using MTT or MTS, suggesting that these dyes give results different from direct measures of ATP or DNA or direct counting of viable cells to determine the antiproliferative effect of green tea polyphenols or other phytochemicals. It is possible that polyphenols may interfere with formazan formation critical to the MTT or MTS methods. Outcomes of the experiments using the methods quantifying ATP and DNA were highly correlated and both of them were consistent with the trypan blue assay.

**Figure 1:**
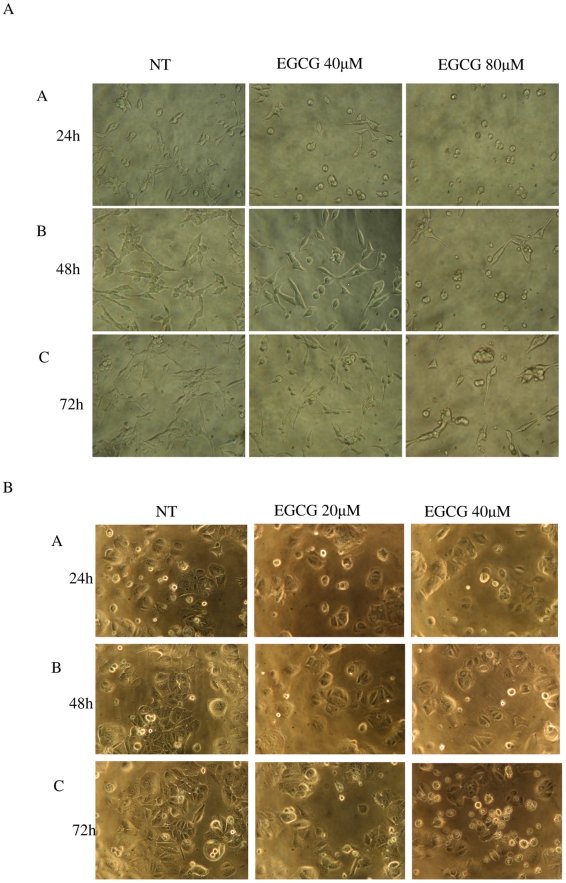
Proliferation of LNCaP cells and MCF-7 cells treated with EGCG. A: LNCaP cells were seeded into 96-well plates at a density of 1×10^4^ per well and treated with the following: vehicle control (NT), EGCG at 40 and 80 µmol/L. B: MCF-7 cells were seeded at a density of 8×10^3^ per well and treated with the following: vehicle control (NT), EGCG at 20 and 40 µmol/L. Pictures were taken by microscope at 24 h (A), 48 h (B), and 72 h (C).

**Figure 2:**
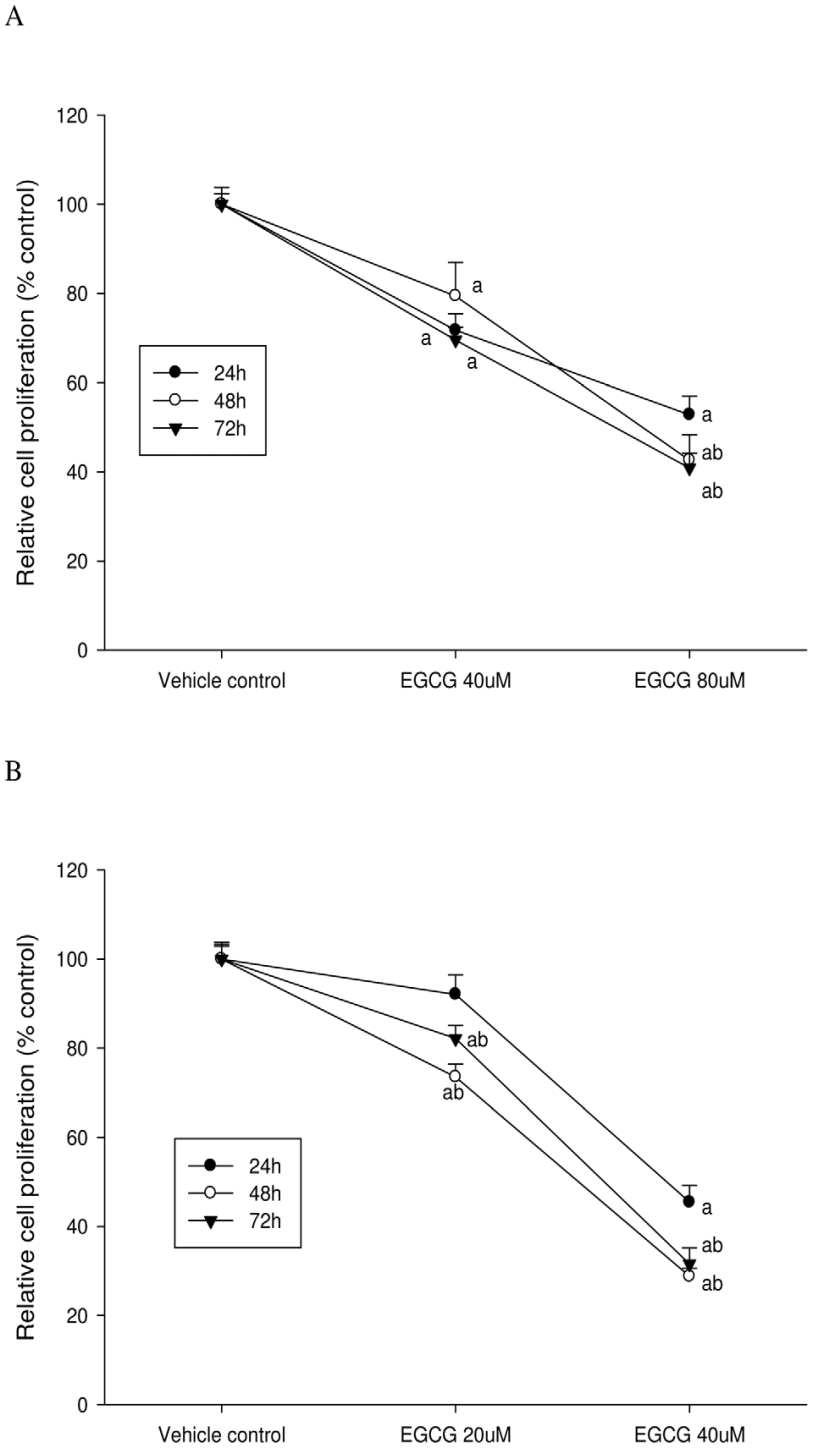
Trypan blue assay was used to determine cell proliferation after EGCG treatment. A: LNCaP cells; B: MCF-7 cells. The superscript letters represent significant difference between groups (P<0.05): ^a^ compared to vehicle control; ^b^ compared to the 24 h value. Error bars represent standard deviation.

**Figure 3:**
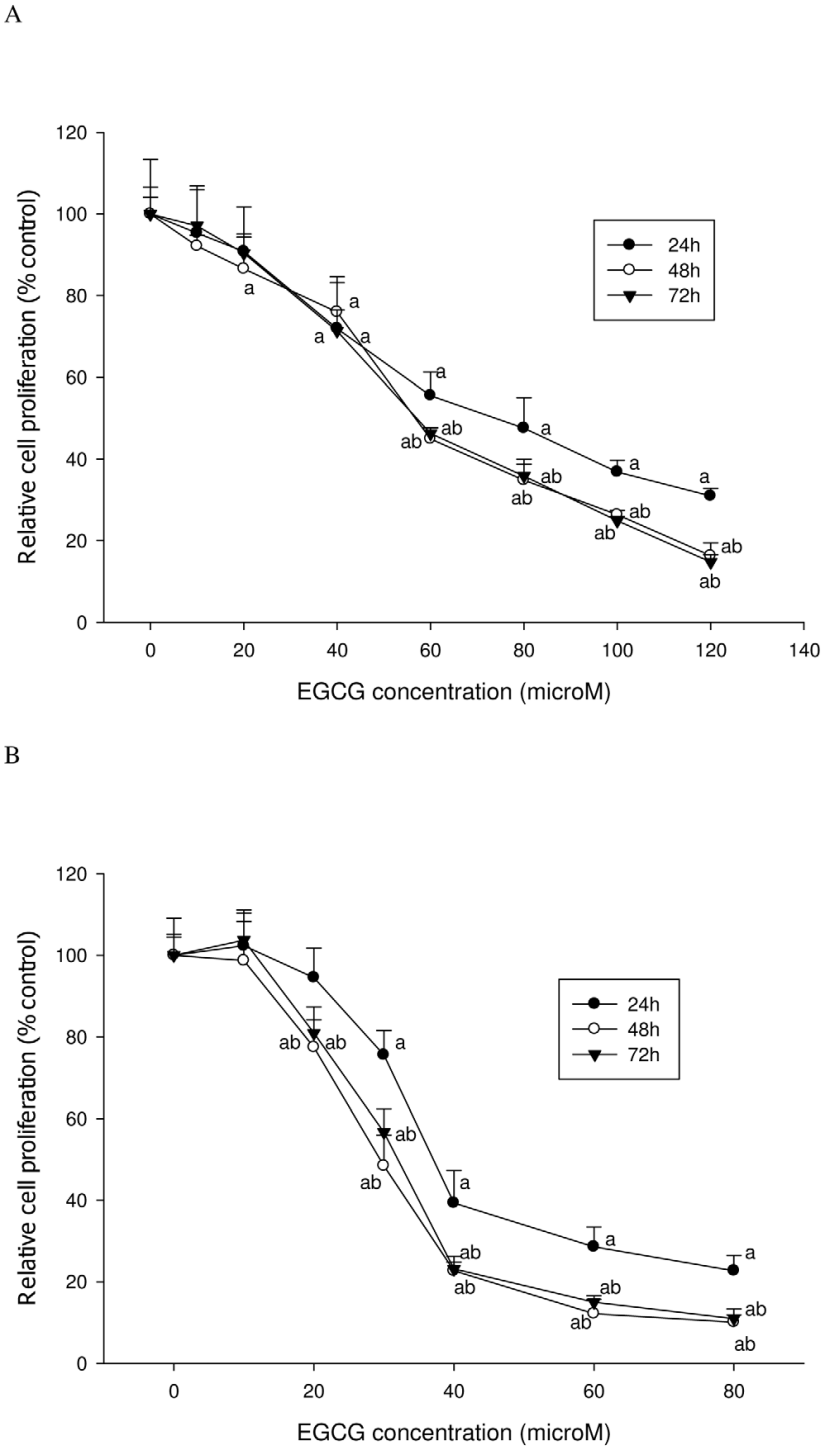
ATP assay was used to determine cell proliferation after EGCG treatment. A: LNCaP cells; B: MCF-7 cells. The superscript letters represent significant difference between groups (P<0.05): ^a^ compared to vehicle control; ^b^ compared to the 24 h value. Error bars represent standard deviation.

**Figure 4:**
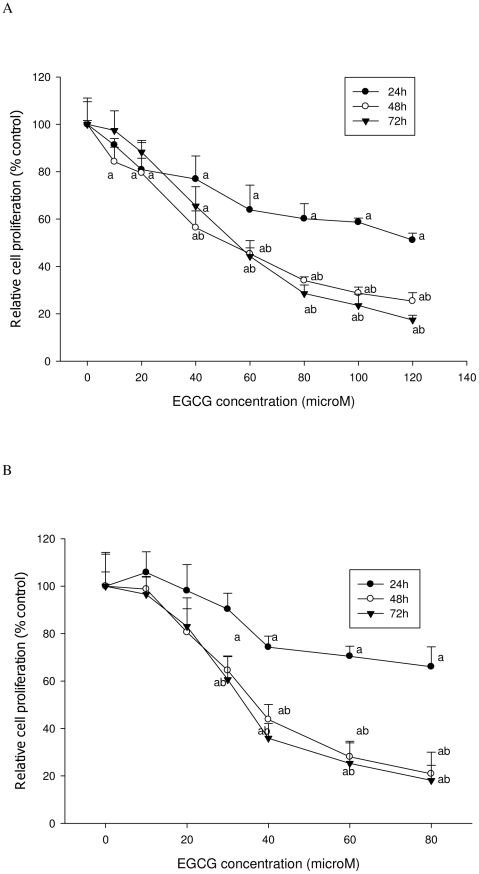
DNA assay was used to determine cell proliferation after EGCG treatment. A: LNCaP cells; B: MCF-7 cells. The superscript letters represent significant difference between groups (P<0.05): ^a^ compared to vehicle control; ^b^ compared to the 24 h value. Error bars represent standard deviation.

**Figure 5:**
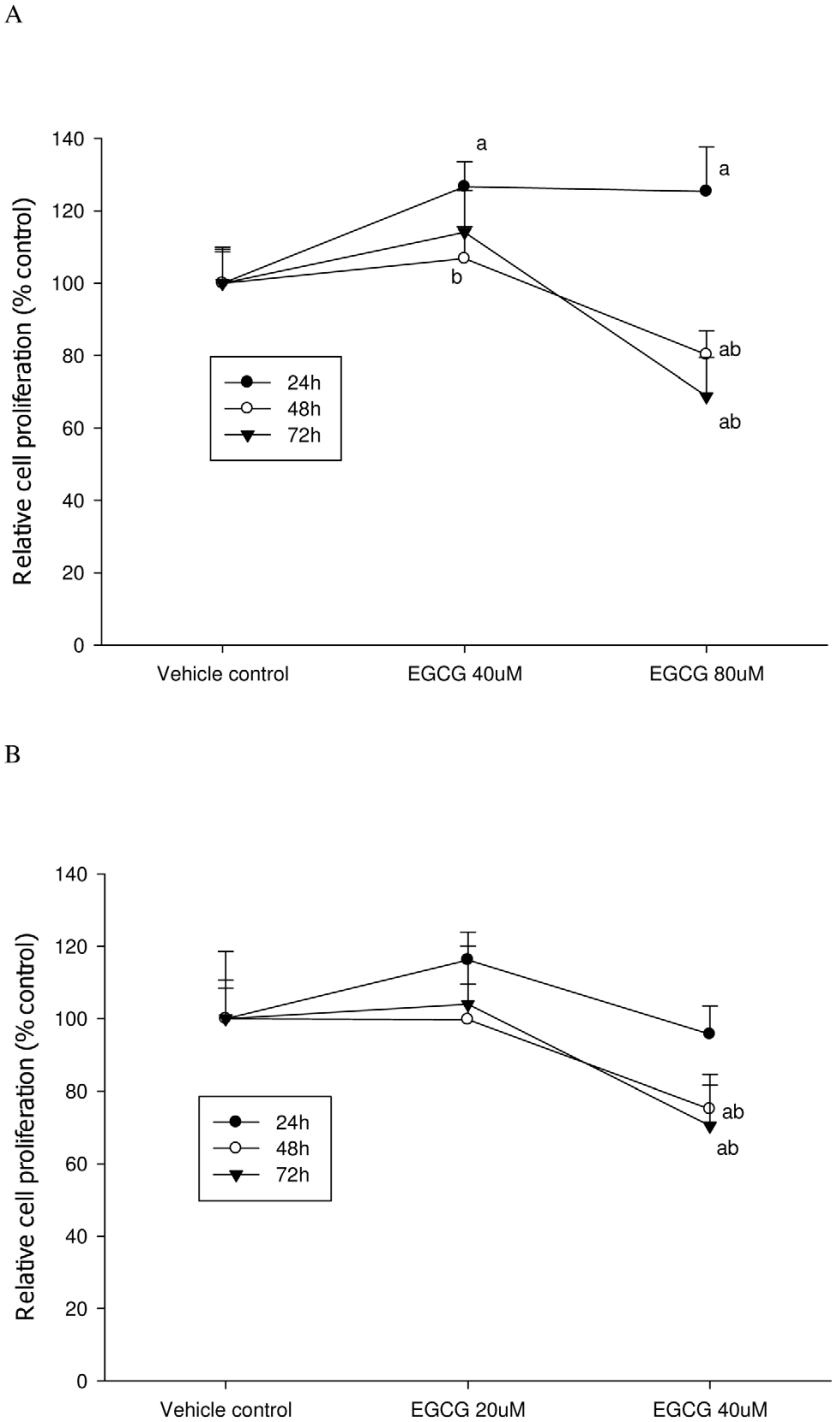
MTT assay was used to determine viability of cells treated with EGCG. A: LNCaP cells; B: MCF-7 cells. The superscript letters represent significant difference between groups (P<0.05): ^a^ compared to vehicle control; ^b^ compared to the 24 h value. Error bars represent standard deviation.

**Figure 6:**
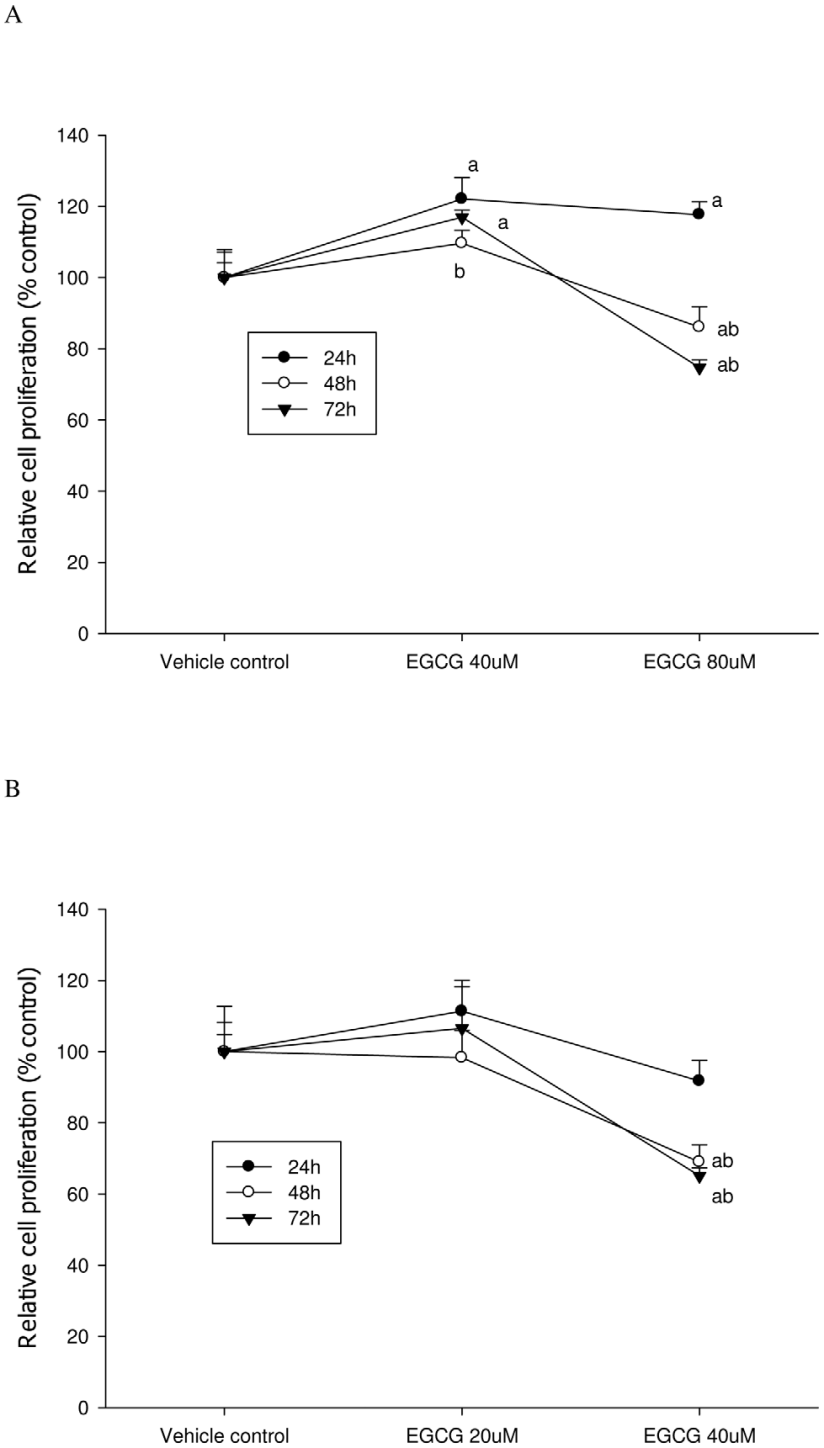
MTS assay was used to determine viability of cells treated with EGCG. A: LNCaP cells; B: MCF-7 cells. The superscript letters represent significant difference between groups (P<0.05): ^a^ compared to vehicle control; ^b^ compared to the 24 h value. Error bars represent standard deviation.

As shown in Figures 1 and 2, EGCG treatment inhibited the proliferation of both LNCaP cells and MCF-7 cells in a dose-dependent manner. Both ATP and DNA assays detected a significant inhibition of LNCaP cell proliferation by 30% compared to the control when cells were treated with 40 µM of EGCG for 72 h (P<0.05) (Figure 3A & 4A); However, using the MTT or MTS methods viability was increased by 15% compared to the control (P<0.05) (Figure 5A & 6A). When LNCaP cells were treated with 80 µM of EGCG for 72 h, cell proliferation was inhibited by 65% using the ATP or DNA methods relative to the control while viability was only inhibited by 30% using the MTT or MTS methods.

Similar results were obtained with MCF-7 cells. A significant inhibition of cell proliferation by 20% was detected using ATP or DNA methods when MCF-7 cells were treated with EGCG at 20 µM for 72 h (Figure 3B & 4B), compared to a 5% increase of viable cell count observed when using MTT or MTS assays (Figure 5B & 6B). When cells were treated with 40 µM of EGCG for 72 h, the inhibition of cell proliferation was around 70% using the ATP or DNA assays relative to the control compared to less than 40% when the MTT or MTS assays were used.

The results derived by using the ATP and DNA methods demonstrated a significant linear relationship (r = 0.95) and IC_50_ values obtained from the ATP and DNA assays were similar: 55 µM of EGCG for LNCaP and 35 µM EGCG for MCF-7 cells at 72 h. However, the IC_50_ values obtained from the MTT or MTS assays were much higher (120 µM of EGCG for LNCaP and 60 µM EGCG for MCF-7 cells at 72 h) when compared to the ATP and DNA methods.

In contrast, when LNCaP cells were treated with apigenin both MTT and MTS assays showed accuracy in measurement of cell growth as demonstrated by a high consistence of the outcome from MTT or MTS assay with that from ATP, DNA, or trypan blue assays (r = 0.96) (Table 1).

**Table 1:** Comparison of different assays to determine the effect of apigenin on LNCaP cell proliferation.

	24 h		48 h		72 h	
Assays	Low dose*	High dose	Low dose	High dose	Low dose	High dose
Trypan blue	84.6±6.7	53.8±7.0^a^	65.7±4.1^a^ ^b^	16.3±2.2^a^ ^b^	58.2±3.9^a^ ^b^	13.4±3.9^a^ ^b^
ATP	84.9±5.8	55.6±7.0^a^	68.8±3.3^a^ ^b^	19.7±2.5^a^ ^b^	60.3±5.8^a^ ^b^	16.4±1.9^a^ ^b^
DNA	83.6±8.9	63.6±6.5^a^	67.4±2.1^a^ ^b^	22.4±0.9^a^ ^b^	59.3±9.2^a^ ^b^	9.7±0.9^a^ ^b^
MTT	81.6±3.5^a^	58.6±2.1^a^	68.2±3.5^a^ ^b^	21.7±2.6^a^ ^b^	64.2±7.0^a^ ^b^	21.7±2.6^a^ ^b^
MTS	85.6±4.2^a^	50.3±2.2^a^	68.6±5.0^a^ ^b^	19.8±1.5^a^ ^b^	64.2±6.0^a^ ^b^	16.4±1.5^a^ ^b^

* LNCaP cells were treated with apigenin at concentrations of 0 (DMSO), 15 µM (low dose), or 30 µM (high dose), and cell growth were assayed at desired time points. Values represent the percentage of viable cells compared to the vehicle (DMSO) control group and are presented in mean ± standard deviation.

a compared to vehicle control, P<0.05;

b compared to the 24 h value, P<0.05.

## Discussion

The present study demonstrated a significant difference in the determination of the antiproliferative activity of EGCG when using the MTT or MTS-based assays by comparison to ATP and DNA-based methods and trypan blue assay. MTT is a tetrazolium salt that is reduced to purple formazan crystals mainly by mitochondrial succinate dehydrogenase [16]. The MTS is an alternative to MTT and the formazan formed from MTS is water-soluble and less toxic [17]. Theoretically, the color intensity of the formazan dye is correlated to the number of viable cells. However, some chemicals or phytochemicals may change the activity of succinate dehydrogenase or interact with MTT directly [13], [18]. In the present study, the MTT and MTS methods did not show an antiproliferative activity using 40 µM of EGCG treatment, and was less sensitive at 80 µM EGCG treatment compared to the ATP- and DNA-based methods. EGCG has been shown to protect against mitochondria injury and greatly increase the activity of succinate dehydrogenase [13], [18]. This may enable the injured cells to reduce MTT or MTS and increase the production of formazan by the same number of cells. In addition, Bruggisser et al. [19] and Wisman et al. [20] found a direct reduction of MTT to formazan by some phenolics including kaempferol and EGCG in the absence of cells, and washing the cells before adding MTT reduced the interference [19]. However, for those cells of less adherence such as LNCaP cells, the washing process may cause loss of cells.

The ATP-based method is highly sensitive, reproducible and simple for the evaluation of cell viability and proliferation [21], [22]. It is based on the quantitation of cellular ATP using the luciferin-luciferase reaction to produce bioluminescence [23]. ATP degrades rapidly in dead cells and declines in injured cells. Therefore, the amount of ATP present in the cells is proportional to the number of viable cells in culture [23]. In the present study, using the ATP-based assay a dose-dependent decrease of viable cell numbers of both LNCaP and MCF-7 cells was observed, and a statistically significant decrease was observed at 40 µM of EGCG treatment of LNCaP cells and 20 µM of EGCG treatment of MCF-7 cells. These results are consistent with the number of cells observed in the microscopic pictures of cell culture plates and the direct cell counting from trypan blue exclusion assay. Similar results were obtained using the DNA-based method, which is based on the measurement of cellular DNA to indicate the relative cell number since cellular DNA content is highly regulated [24]. In the DNA-based assay kit used, the fluorescent dye binds to cellular nucleic acid and exhibits strong fluorescence with an excitation wavelength of 485 nm. Both ATP- and DNA-based methods were able to detect a linear range from less than 100 to 100,000 cells/well [21], [24]. The values from these two assays showed a significant correlation in the present study. However, using the DNA-based method the medium has to be removed before adding the reagent, which may cause loss of cells that are not well attached to the culture plate.

Regarding the possibility of the interference caused by cell density, we seeded LNCaP cells at different densities: 3×10^3^ per well and 6×10^3^ per well on 96-well plates, however, a similar outcome was observed compared to seeding cells at 1×10^4^ per well using the five assays (data not shown). In addition, we examined the possible interferences of different cell types with the assays using another flavonoid apigenin as a control compound. Eventually all the assays performed well to assess the inhibition of cell proliferation by apigenin treatment, which further supports that the interference with MTT and MTS assays was caused by EGCG.

In summary, MTT and MTS-based methods resulted in an underestimation of the anti-proliferative effects of EGCG probably due to the increased activity of mitochondrial dehydrogenase in response to EGCG treatment and the intrinsic potential of EGCG to reduce MTT and MTS and increase the formation of formazan. The ATP- and DNA-based methods were highly correlated and both demonstrated antiproliferative activity of EGCG consistently. These results demonstrate the need for careful evaluation of the methods used for the in vitro evaluation of cell proliferation depending on the chemical nature of botanical supplements to be evaluated.
